# Central diabetes insipidus unveiled by glucocorticoid therapy in a patient with an empty sella: A case report and literature review

**DOI:** 10.1097/MD.0000000000022939

**Published:** 2020-10-23

**Authors:** Lei-Yi Yang, Sang Lin, Qi-Bing Xie, Geng Yin

**Affiliations:** Department of Rheumatology and Immunology, West China Hospital, Sichuan University, Chengdu, Sichuan, China.

**Keywords:** central diabetes insipidus, glucocorticoid, empty sella, Guillain–Barré syndrome

## Abstract

**Rationale:**

Some diseases contribute to hypopituitarism without clinical manifestations and the glucocorticoid therapy may unveil central diabetes insipidus. The condition is rare and usually causes problems for clinical physicians.

**Patient concerns:**

A 59-year-old woman presented to our hospital due to facial numbness and persistent eyelid heaviness.

**Diagnosis:**

Physical examination and cerebrospinal fluid examination supported a diagnosis of Guillain–Barré syndrome. Magnetic resonance imaging showed an empty sella. Hormone test indicated hypopituitarism.

**Interventions:**

The patient received intravenous immunoglobulin and glucocorticoid. Central diabetes insipidus appeared after 20 days. Subsequently, the patient was prescribed 1-desamino-8-D-arginine vasopressin and prednisone.

**Outcomes:**

During 6 months’ follow-up, the patient's urine output was gradually reduced to normal level.

**Lessons:**

This case indicated that hypopituitarism may be caused by an empty sella and be masked by adrenal insufficiency. Central diabetes insipidus may present after glucocorticoid therapy.

## 1 Introduction

Empty sella is one of the causes of hypopituitarism, and refers to an intrasellar herniation of the subarachnoid space through a congenital defect in the diaphragm sellae or pituitary involution.^[1]^ Central diabetes insipidus may be masked by adrenal insufficiency and uncovered by subsequent steroid therapy, due to involvement of arginine vasopressin (AVP)-dependent and AVP-independent mechanisms.^[2]^ Here, we report a rare case of a patient with empty sella who developed central diabetes insipidus following glucocorticoid (GC) administration.

## 2 Case report

**Figure 1:**
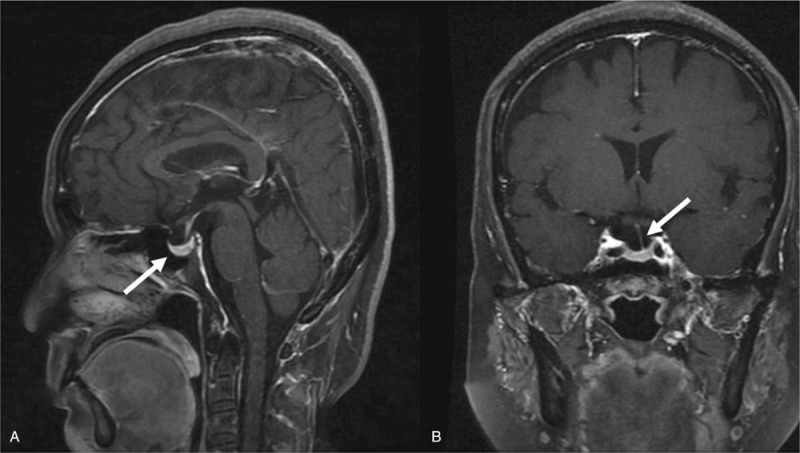
Magnetic resonance images. (A) T1-weighted image in sagittal section view shows that the pituitary gland is pressed downwards, and the thickness is about 0.4 cm (white arrow). (B) T1-weighted image in coronal section view shows that the pituitary stalk is shifted to the left (white arrow).

A 59-year-old woman presented to the Neurology Department in July 2019 with a history of numbness of the face and persistent eyelid heaviness for 10 days. Physical examination revealed an attenuated tendon reflex, negative pyramidal sign, and progressively worsening symmetrical weakness of the extremities. Her family and past histories were unremarkable. Magnetic resonance imaging of the brain showed an empty sella (Fig. 1) and electrophysiologic evidence of neurogenic injury in the extremities was also discovered. Hormone tests demonstrated decreased levels of adrenocorticotropic hormone (ACTH), serum free triiodothyronine, and free thyroxine, thus indicating hypopituitarism. Cerebrospinal fluid (CSF) examination revealed no abnormal findings. According to the diagnostic criteria for Guillain-Barré syndrome (GBS) proposed by the National Institute of Neurological Disorders and Stroke in 1978,^[3,4]^ the symptoms of progressively worsening symmetrical weakness of the extremities along with a reduced tendon reflex in this patient were consistent with a diagnosis of GBS. According to a study by Asbury et al,^[3]^ unremarkable CSF findings were not an exclusion criteria, since CSF examination in some patients did not reveal typical albuminocytologic dissociation within 1 to 10 weeks from the onset of the symptoms. Once the diagnosis was confirmed, the patient received intravenous immunoglobulin (20g × 5 days), methylprednisolone (1000mg × 5 days and then 500mg × 5 days), and dosage-tapered oral prednisone. Her 24-hour urine output increased (4000–10000 ml/day) after 20 days, and urinalysis showed low specific gravity (SG) of the urine (1.005) along with normal renal function. Subsequently, a high dosage of 1-desamino-8-D-arginine vasopressin (DDAVP) was administered which decreased the 24-hour urine output. In order to identify the etiology, she underwent an 8-hour fluid deprivation-vasopressin test (Table 1). During the test, symptoms of hypovolemia were observed; however, there was little increase in the urinary osmotic pressure and the urine SG remained below 1.010, suggesting a deficiency of AVP. On administration of 5 U of exogenous AVP, the urinary SG increased rapidly with doubling of the urine osmolality, and the ratio of urinary osmotic pressure to plasma osmotic pressure was more than 1.0, indicating that the patient responded appropriately to AVP. Thus, these findings supported a diagnosis of central diabetes insipidus (CDI). Although an increase in the serum sodium is a feature of CDI, it may be normal at the time of clinical diagnosis.^[5]^ Consequently, the patient was prescribed daily DDAVP and prednisone. After six months, her urine output reduced to normal and the weakness of the extremities disappeared.

**Table 1:**
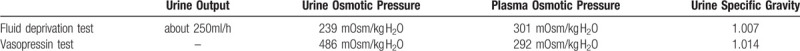
Results of an 8-hour fluid deprivation-vasopressin test.

## 3 Discussion

CDI is characterized by a failure to maximally concentrate the urine along with a deficiency of AVP.^[6]^ Occasionally, it may be masked by adrenal insufficiency caused by hypopituitarism and uncovered by corticosteroid therapy. The phenomenon of polyuria due to CDI masked by a GC deficiency has been described in other case reports.^[2,7–22]^ The reported patients had hypopituitarism attributable to different diseases and did not manifest polyuria in the early stage; however, the typical manifestations of CDI appeared after the administration of GCs (Table 2).

**Table 2:**
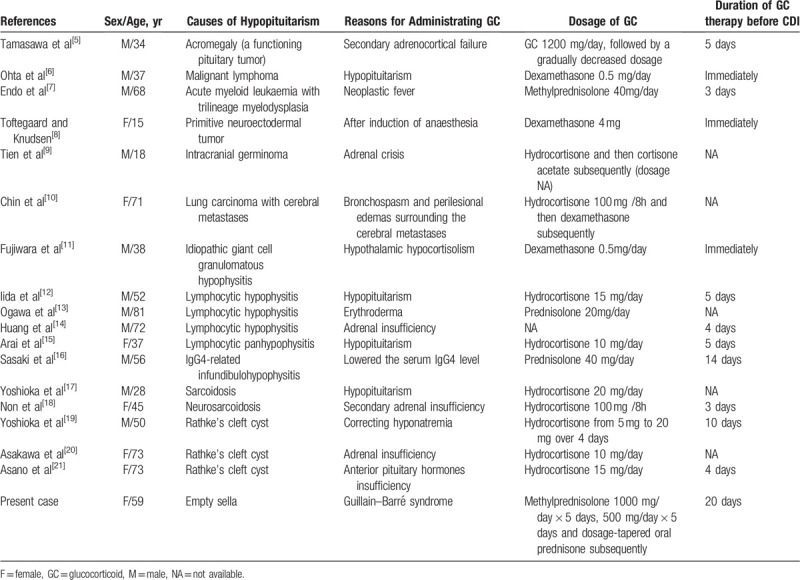
A summary of previously published studies of CDI followed by GC therapy in patients with hypopituitarism.

The mechanisms of CDI and hypopituitarism are inter-connected. It is difficult to identify CDI before starting GC therapy in patients with hypopituitarism because they do not manifest the characteristic symptoms of CDI. In the present case, hypopituitarism was caused by an empty sella. Empty sella compresses the pituitary and causes pituitary hormone deficiency in 8% to 60% of the patients,^[23]^ particularly leading to a defective anterior pituitary function.^[24]^ There are also a few case reports of posterior empty sella in children presenting with CDI, and in these cases the symptoms may be seen in the early years. Nevertheless, the pathogenic mechanisms remain unclear.^[25]^

**Figure 2:**
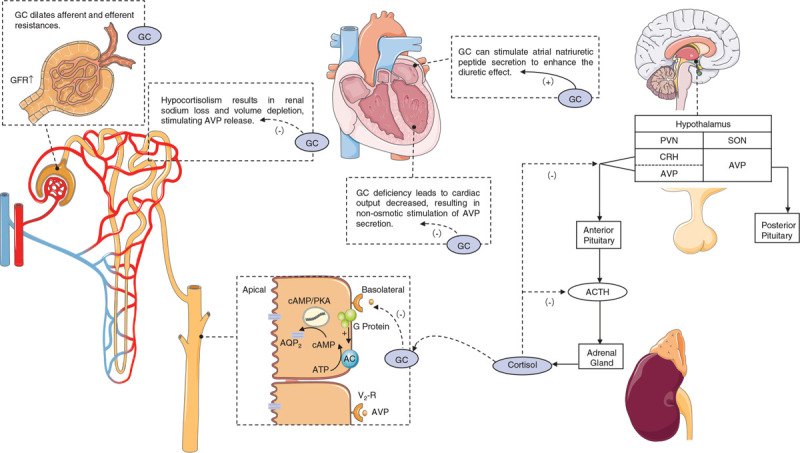
Schematic figure demonstrating the reasons for central diabetes insipidus after glucocorticoid administration.

AVP, also known as antidiuretic hormone, is synthesized by both the supraoptic and paraventricular nuclei in the hypothalamus. AVP is transported within secretory granules through the supraoptic-hypophyseal tract to the posterior pituitary gland, where the granules are stored and secreted on osmotic stimulation.^[26]^ The mechanisms of GC deficiency that impair renal water excretion are both AVP-dependent and AVP-independent. First, cortisol induces relative resistance of the V2 receptor to AVP, which decreases the translocation of type 2 aquaporins for water reabsorption. Thus, in case of GC deficiency, the effects of AVP are amplified.^[27]^ Second, corticotrophin-releasing hormone (CRH) neurons in the paraventricular nucleus stimulate the release of ACTH and AVP. Additionally, CRH is upregulated in states of GC deficiency and, thus, causes release of AVP.^[2]^ Third, hypocortisolism results in renal sodium loss and volume depletion, which are potent stimulators for appropriately increasing the release of AVP. Lastly, GC deficiency also leads to a decrease in the stroke volume and cardiac output, resulting in non-osmotic stimulation of AVP secretion.^[28]^ Green et al found that adrenalectomised rats with hereditary diabetes insipidus showed decreased free water excretion, which was later rectified with GC replacement.^[29]^ This indicates mechanisms that are independent of AVP. GC dilates both afferent and efferent resistances resulting in an increase in the glomerular filtration rate (GFR); consequently, the rate of water flow into the nephron increases.^[30]^ Furthermore, an increase in the water permeability of the distal tubules was found in adrenalectomized Brattleboro rats, suggesting a direct action of GC on renal tubules.^[31]^ Hayamizu et al reported that GC can stimulate atrial natriuretic peptide secretion to enhance the diuretic effects.^[32]^ As such, ameliorating GC deficiency can lead to a reversal of these compensatory mechanisms, and diabetes insipidus ensues (summarized in Fig. 2). Moreover, the timing of GC deficiency may play a role in the mechanisms of CDI. Linas et al studied the role of AVP in defective water excretion in rats with diabetes insipidus and demonstrated that an AVP-dependent impairment in water excretion after 24 hours of GC deficiency played a role. Additionally, an AVP-independent factor was observed after 2 weeks of GC deficiency.^[33]^

We further hypothesized that the dosage and duration of GC administration played a role in the development of CDI. Unfortunately, since the data concerning dosage and duration of GC in some previous studies were not available (summarized in Table 2), the statistical results may be biased. We can conclude that either the maintenance dosage or stress dosage of GC can unmask CDI. However, no cases have been reported in which CDI was induced only when greater than the maintenance dosage of GCs was given.

In conclusion, empty sella is one of the causes of hypopituitarism, and GC replacement in patients with hypopituitarism can unveil CDI. Our report is the first to describe the two mechanisms mentioned above seen together in a patient with an empty sella. GC deficiency may lead to impaired water excretion, which is mediated by AVP-dependent and AVP-independent mechanisms. Therefore, attention should be paid when hypopituitarism is encountered in the clinic as CDI may present on administration of GC.

## Author contributions

**Conceptualization:** Lei-Yi Yang

**Data curation:** Sang Lin

**Methodology:** Geng Yin

**Supervision:** Qi-Bing Xie

**Writing – original draft:** Lei-Yi Yang

**Writing – review & editing:** Geng Yin
